# Supplementary material for: Understanding potential-dependent competition between electrocatalytic dinitrogen and proton reduction reactions
Source: Nat Commun. 2021 Jul 16;12:4353. doi: 10.1038/s41467-021-24539-1 (PMC8285508; doi:10.1038/s41467-021-24539-1)
Supplement: Supplementary file 1 — Supplementary Information [file 41467_2021_24539_MOESM1_ESM.pdf]

Supporting Information

Understanding Potential-dependent Competition Between Electrocatalytic Dinitrogen and Proton Reduction Reactions

Changhyeok Choi[1], Geun Ho Gu[1], Juhwan Noh[1], Hyun S. Park[2], and Yousung Jung[1]*

[1]Department of Chemical and Biomolecular Engineering (BK21 four) Korea Advanced Institute of Science and Technology (KAIST) 291 Daehak-ro, Yuseong-gu, Daejeon 34141, Republic of Korea

[2]Center for Hydrogen and Fuel Cell Research, Korea Institute of Science and Technology (KIST), Seoul 02792, Republic of Korea

Supplementary Note 1. General trend of the potential-dependent NH_3 yield rate

Supplementary Table 1 | Summary of the reported catalysts for NRR at low temperature. The maximum yield rate, maximum faradaic efficiency (FE) and the electrode potential (*U*) at the maximum yield rate are shown.

Catalyst	Electrolyte	NH_3 yield	FE (%)	*U* vs. RHE (V)
Rh nanosheet[1]	0.1 M KOH	23.88 μg $h^{-1}mg_{cat}^{-1}$	0.217	-0.2
(110)-oriented Mo[2]	0.5 M H_2SO_4	3.09×10^{-11} mol s^{-1} cm^{-2}	0.72	-0.49
Fe/Fe_3O_4[3]	0.1 M PBS	0.19 μg cm^{-2} h^{-1}	8.29	-0.3
Ru NP[4]	0.01 M HCl	5.5 mg h^{-1} m^{-2}	5.4	-0.1
THH Au nanorods[5]	0.1 M KOH	1.648 μg h^{-1} cm^{-2}	4.02	-0.2
Ru@NC[6]	0.1 M HCl	3.665 mg $h^{-1}mg_{Ru}^{-1}$	~ 9	-0.21
VN nanowire[7]	0.1 M HCl	2.48×10^{-10} mol s^{-1} cm^{-2}	3.58	-0.3
VN[8]	0.05 M H_2SO_4	3.3×10^{-10} mol s^{-1} cm^{-2}	6.0	-0.2
Mo_2N[9]	0.1 M HCl	78.4 μg h^{-1} $mg_{cat.}^{-1}$	4.5	-0.3
1T-MoS_2 NDs/g-C_3N_4[10]	0.1 M HCl	29.97 μg h^{-1} $mg_{cat.}^{-1}$	20.48	-0.3
Fe-N/C-CNT[11]	0.1 M KOH	34.83 μg h^{-1} $mg_{cat.}^{-1}$	9.28	-0.2
Fe-N-C[12]	0.1 M KOH	7.48 μg h^{-1} mg^{-1}	56.55	0.0
ISAS-Fe/NC[13]	0.1 M PBS	62.9 μg h^{-1} $mg_{cat.}^{-1}$	18.6	-0.40
SA-Ag/NC[14]	0.1 M HCl	270.9 μg h^{-1} mg^{-1}	21.9	-0.65
Mo-doped FeS_2[15]	0.1 M KOH	26.15 μg h^{-1} mg^{-1}	14.41	-0.2
Defective TiO_2[16]	0.5 M H_2SO_4	3.0 μg h^{-1} $mg_{cat.}^{-1}$	6.5	-0.12
ZIF-derived carbon[17]	0.1 M KOH	3.4×10^{-6} mol cm^{-2} h^{-1}	10.2	-0.3
B-doped graphene[18]	0.05 M H_2SO_4	9.8 μg h^{-1} cm^{-2}	10.8	-0.5
DrGO[19]	0.1 M HCl	7.3 μg $h^{-1}mg^{-1}$	22.0	-0.116

Supplementary Note 2. The potential-dependent energetics at different pH.

The formation energy of reaction intermediates in Fig. 2 and HER is represented as below in the computational hydrogen electrode (CHE) model.

$$\Delta G(*\mathrm{H}) = G(*\mathrm{H}) - [G(*) + G(\mathrm{H}^+ + \mathrm{e}^-)] \quad \textbf{(1)}$$

$$\Delta G(*\mathrm{N_2}) = G(*\mathrm{N_2}) - [G(*) + G(\mathrm{N_{2(g)}})] \quad \textbf{(2)}$$

$$\Delta G(*\mathrm{NNH}) = G(*\mathrm{NNH}) - [G(*) + G(\mathrm{N_{2(g)}}) + G(\mathrm{H}^+ + \mathrm{e}^-)] \quad \textbf{(3)}$$

$$\Delta G(*\mathrm{NHNH}) = G(*\mathrm{NHNH}) - [G(*) + G(\mathrm{N_{2(g)}}) + 2G(\mathrm{H}^+ + \mathrm{e}^-)] \quad \textbf{(4)}$$

$$\Delta G(*\mathrm{NNH_2}) = G(*\mathrm{NNH_2}) - [G(*) + G(\mathrm{N_{2(g)}}) + 2G(\mathrm{H}^+ + \mathrm{e}^-)] \quad \textbf{(5)}$$

$$\Delta G(*\mathrm{NHNH_2}) = G(*\mathrm{NNH}) - [G(*) + G(\mathrm{N_{2(g)}}) + 3G(\mathrm{H}^+ + \mathrm{e}^-)] \quad \textbf{(6)}$$

$$\Delta G(*\mathrm{NH_2NH_2}) = G(*\mathrm{NNH}) - [G(*) + G(\mathrm{N_{2(g)}}) + 4G(\mathrm{H}^+ + \mathrm{e}^-)] \quad \textbf{(7)}$$

$$\Delta G(*\mathrm{N}) = G(*\mathrm{N}) - [G(*) + 1/2\ G(\mathrm{N_{2(g)}})] \quad \textbf{(8)}$$

$$\Delta G(*\mathrm{NH}) = G(*\mathrm{NH}) - [G(*) + 1/2\ G(\mathrm{N_{2(g)}}) + G(\mathrm{H}^+ + \mathrm{e}^-)] \quad \textbf{(9)}$$

$$\Delta G(*\mathrm{NH_2}) = G(*\mathrm{NH_2}) - [G(*) + 1/2\ G(\mathrm{N_{2(g)}}) + 2G(\mathrm{H}^+ + \mathrm{e}^-)] \quad \textbf{(10)}$$

$$\Delta G(*\mathrm{NH_3}) = G(*\mathrm{NH_3}) - [G(*) + 1/2\ G(\mathrm{N_{2(g)}}) + 3G(\mathrm{H}^+ + \mathrm{e}^-)] \quad \textbf{(11)}$$

Contrary to the computational hydrogen electrode (CHE) model, the number of transferred electrons are not integer in the constant electrode potential (CEP) model. To calculate $\Delta G(*\mathrm{N_xH_y})$ in the CEP model, we decoupled the chemical potential of proton ($\mu(\mathrm{H}^+)$) from that of the proton-electron pair ($\mu(\mathrm{H}^+ + \mathrm{e}^-)$) by using the CHE relation[20-22]. When the 1 bar of gas H_2 is equilibrium with the pair of proton and electron at pH = 0 (i.e. $1/2\ \mathrm{H_2} \leftrightarrow \mathrm{H}^+ + \mathrm{e}^-$), 0 V (vs. SHE) is defined. At 0 V (vs. SHE), the chemical potential of a pair of proton and electron is defined by **(12)**, which is the well-known CHE model[22].

$$\mu(\mathrm{H}^+ + \mathrm{e}^-) = \frac{1}{2}\mu(\mathrm{H_2}) \quad \textbf{(12)}$$

The chemical potential of proton is decoupled from the **(12)**.

$$\mu(\mathrm{H}^+) = \frac{1}{2}\mu(\mathrm{H_2}) - \mu(\mathrm{e}^-) \quad \textbf{(13)}$$

From the definition of electrode potential (U) as in **(14)**, $\mu(\mathrm{e}^-)$ is equal to the $-\Phi_{\mathrm{SHE}}$ at 0 V (vs. SHE). Here, Φ_{SHE} represents the absolute potential of SHE.

$$U\ (\mathrm{V\ vs.\ SHE}) = \frac{-\mu(\mathrm{e}^-)-\Phi_{\mathrm{SHE}}}{e}\ \mathbf{(14)}$$

Consequently, the $\mu(\mathrm{H}^+)$ can be related to $\mu(\mathrm{H_2})$ as **(15)** by replacing $\mu(\mathrm{e}^-)$ to $-\Phi_{\mathrm{SHE}}$ in **(13)**.

$$\mu(\mathrm{H}^+) = \frac{1}{2}\mu(\mathrm{H_2}) + \Phi_{\mathrm{SHE}}\ \mathbf{(15)}$$

When pH $\neq$ 0, the effect of pH on $\mu(\mathrm{H}^+)$ is included as **(16)**.

$$\mu(\mathrm{H}^+) = \frac{1}{2}\mu(\mathrm{H_2}) + \Phi_{\mathrm{SHE}} - 0.059\mathrm{pH}\ \mathbf{(16)}$$

Here, we used 4.43 eV for Φ_{SHE}[23,24]. In this study, the electrode potential (*U*) is represented as SHE (pH = 0) or RHE scale. When pH $\neq$ 0, we convert the SHE scale electrode potential into the RHE scale by **(17)**.

$$U\ (\mathrm{vs.\ RHE}) = U\ (\mathrm{vs.\ SHE}) + 0.059\ \mathrm{pH}\ \mathbf{(17)}$$

To calculate activation energy of the proton-coupled electron transfer (PCET) step at the interface, we used solvated H_3O^+ or H_2O as a proton donor. In acidic conditions (pH = 0), we used solvated H_3O^+ as a proton donor. Before proceeding the electrochemical reaction at the surface-electrolyte interface, the proton (H^+) must be brought from somewhere in the bulk electrolyte to near the surface (Helmholtz plane). This step is known as a proton-shuttling step. The proton-shuttling accompanies fractional charge transfer and reaction energy. Thus, we chose reference state as * + H^+(bulk) rather than * + H^+(HP) in calculating G_a(*H) and ΔG(*H), where the H^+(bulk) and H^+(HP) represent H^+ in bulk electrolyte and H^+ in Helmholtz plane, respectively[20,25]. In the cases of calculating G_a(*N_2→*NNH) and ΔG(*N_2→*NNH), we chose *N_2 + H^+(bulk) as a reference state, where *N_2 indicates adsorbed N_2 (Supplementary Fig. 1). Here, the chemical potential of H^+(bulk) is equal to the $\mu(\mathrm{H}^+)$ in **(15)**.

In proton transfer reaction by H_2O (neutral and alkaline conditions), the solvated H_2O in the Helmholtz plane (HP) is considered as a reference state and a proton donor. The accurate calculation of solvation energy of OH^- in implicit solvation model is still challenging[26], and hence, we further correct the electronic energy of OH^- [27]. For electrochemical reaction of $2H_2O + 2e^- \rightarrow H_2(g) + 2OH^-$ (bulk), we compare the calculated free energy with the experimentally determined free energy (2*U* + 1.652 eV at standard state). We considered solvated OH^- above the 3 Å from the equilibrium position in the OH^-

(HP) as a OH^-(OHP) (Supplementary Fig. 2). Previous study showed that the sum of the net charge of H_2O layer including one OH^- converges at -1 e^- when the electrolyte layer is apart from more than 2 Å from the equilibrium position in the HP[28]. Consequently, we correct -0.702 eV for solvated OH^-.

The formation energy (or activation energy) at certain U can be expressed as below.

$$\Delta G(U) \text{ (or } G_a(U)) = \alpha U + \beta \quad \textbf{(18)}$$

The α and β represents the amount of charge transfer in the reaction and ΔG (or G_a) at 0 V, respectively. We listed all of the potential-dependent energetics used in the MKM (Supplementary Table 2). A transition state (TS) is found for all proton-coupled electron transfer (PCET) step. After the OH^- correction, activation energy becomes negative for several elementary reactions. These reactions are highly exothermic (ΔG < -0.4 eV even at 0 V), thus we set these activation energy as 0 eV. Here, the G_a(A→B) represents the activation energy for reaction of A + H_2O → B + OH^- (or A + H^+ → B).

Due to the potential-dependent charge transfer resulted from the change of geometry (Supplementary Fig. 7), the slope obtained from a wide range of potential (e.g. ~ 1 V) may results in the less accurate energy prediction (Fig. 4). Thus, we chose a range of 0.5 V to obtain α and β. The α and β are obtained by linear regression of ΔG (or G_a) at three different U. We used U = 0, -0.23 and -0.5 V (vs. RHE at pH = 13) for alkaline conditions, U = -0.34, -0.58 and -0.84 V (vs. RHE at pH = 7.2) and U = 0, -0.25 and -0.5 V (vs. RHE at pH = 0) for acidic codntions.

Under alkaline and neutral condition, G_a of all PCET steps were calculated. Since experiment in acidic condition has not been reported, we only calculated the G_a(*N_2→*NNH), the RDS of NRR, to verify the premature decrease of NRR activity in acidic condition. We find that the MKM results do not change significantly whether all G_a are included or only the G_a(*N_2→*NNH) is included (Supplementary Fig. 8a). As considering only the G_a(*N_2→*NNH) is sufficient to estimate the NRR activity, we calculated ΔG without explicit water layer for other elementary steps of NRR to save the computational cost. We find that the mean absolute difference of ΔG with and without explicit water layer is 0.10 eV (Supplementary Fig. 8b), which is not expected to affect the overall NRR kinetics signficiantly.

In NRR experiments, NH_3 is obatined in aqueous phase (solvated NH_3 or NH_4^+). Thus, we chose $NH_{3(aq)}$ rathern than $NH_{3(g)}$ as a final product. The free energy of $NH_{3(aq)}$ was calculated using the free energy of $NH_{3(g)}$ and the vapor pressure of 1 M of aqueous ammonia solution[29]. In other gas molecules (N_2 and H_2), free energies were calculated at 1 atm.

Supplementary Table 2 | List of the potential-dependent energetics at different pH. The *α* and *β* are represented in the RHE scale at each pH value. Activation energy for adsorption/desorption of N_2 and NH_3 is not considered. The unit of *β* is eV.

Reactions	Alkaline (pH = 13) *α*	Alkaline (pH = 13) *β*	Neutral (pH = 7.2) *α*	Neutral (pH = 7.2) *β*	Acidic (pH = 0) *α*	Acidic (pH = 0) *β*
G_a(*H)	0.96	1.49	0.96	1.11	0.72	0.73
ΔG(*H)	1.34	0.44	1.34	0.56	1.12	0.63
G_a(*H→H_2)	0.56	0.39	0.56	0.59	0.43	0.26
ΔG($H_{2(g)}$)	2	0	2	0	2	0
ΔG(*N_2)	0.30	0.31	0.30	0.41	0.18	0.40
G_a(*N_2→*NNH)	0.76	1.50	0.76	1.76	0.33	1.16
ΔG(*NNH)	1.40	1.66	1.40	1.80	0.33	1.44
G_a(*NNH→*NNH_2)	0.54	0.26	0.54	0.44		
ΔG(*NNH_2)	2.06	1.88	2.06	1.90	1.74	1.63
G_a(*NNH_2→*N+$NH_{3(aq)}$)	0.72	0.41	0.72	0.66		
ΔG(*N)	0.43	2.06	0.43	2.21	-0.06	2.06
G_a(*N→*NH)	0.73	0.06	0.73	0.31		
ΔG(*NH)	1.57	1.54	1.57	1.74	1.18	1.80
G_a(*NH→*NH_2)[a)]	0.22	-0.46	0.22	-0.39		
ΔG(*NH_2)	2.25	0.48	2.25	0.57	2.10	0.62
G_a(*NH_2→*NH_3)[a)]	0.14	-0.35	0.14	-0.30		
ΔG(*NH_3)	2.83	0.05	2.83	-0.01	2.59	-0.51
G_a(*NNH→*NHNH)	0.53	0.25	0.53	0.44		
ΔG(*NHNH)	2.09	1.58	2.09	1.61	1.86	1.69
G_a(*NHNH→*$NHNH_2$)	0.24	0.29	0.24	0.37		
ΔG(*$NHNH_2$)	3.03	2.01	3.03	2.02	2.28	1.38
G_a(*$NHNH_2$→*NH_2NH_2)[a)]	0.30	-0.01	0.30	0.10		
ΔG(*NH_2NH_2)	3.83	1.63	3.83	1.57	3.63	1.28
G_a(*NH_2NH_2→*NH_2+$NH_{3(aq)}$[a)]	0.29	-0.18	0.29	-0.08		
G_a(*NNH_2→*$NHNH_2$)	0.31	0.12	0.31	0.23		
G_a(*$NHNH_2$→*NH+$NH_{3(aq)}$)[a)]	0.56	-0.18	0.56	0.01		
ΔG($NH_{3(aq)}$)	3	-0.31	3	-0.31	3	-0.31

[a)] G_a at 0 V becomes negative after OH^- correction.

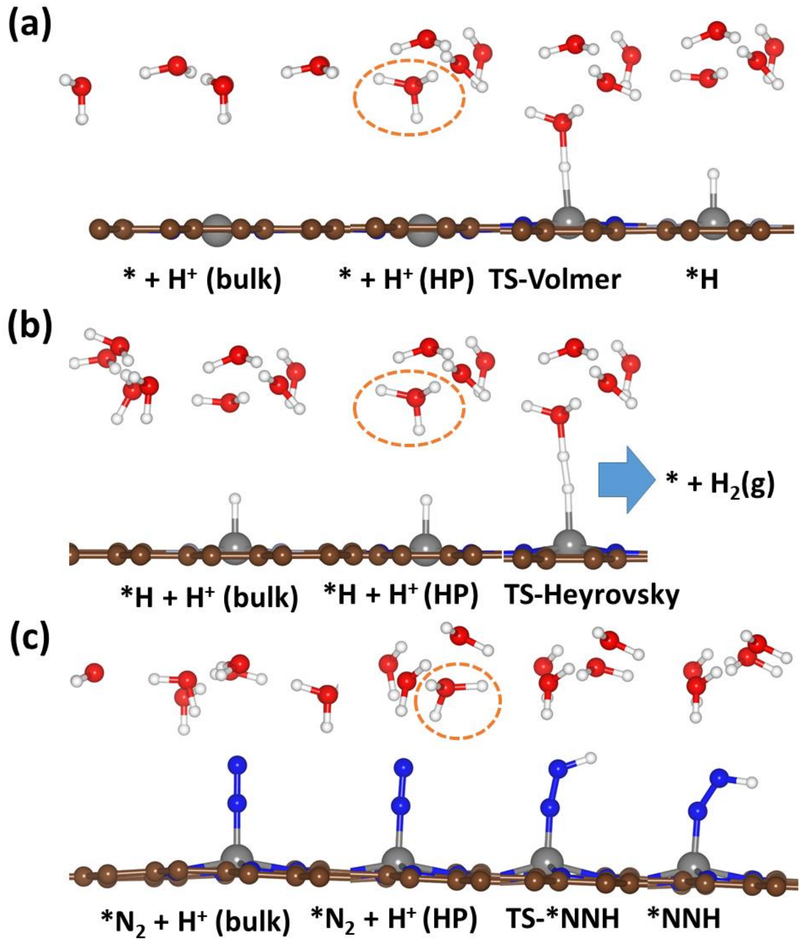

Supplementary Fig. 1 | The optimized geometries of reaction intermediates for HER and *NNH formation by using H_3O^+. (a) The Volmer reaction (* + H^+ (bulk) → *H), (b) the Heyrovsky reaction (*H + H^+ (bulk) → * + H_2(g)) and (c) *NNH formation (*N_2 + H^+ (bulk) → *NNH). The H^+(HP), transferred to surface or adsorbate is highlighted in a yellow circle.

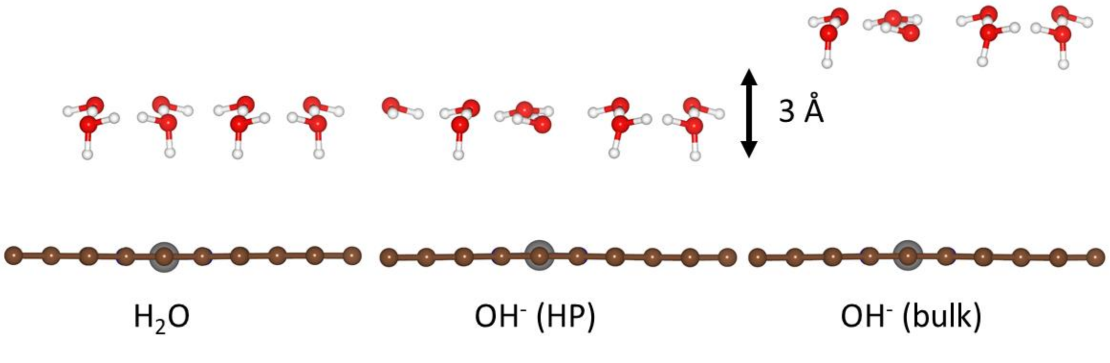

Supplementary Fig. 2 | The optimized structures of solvated H_2O, OH^- in HP and OH^- in bulk. The OH^-(bulk) is apart from the OH^-(HP) by 3 Å in c-axis.

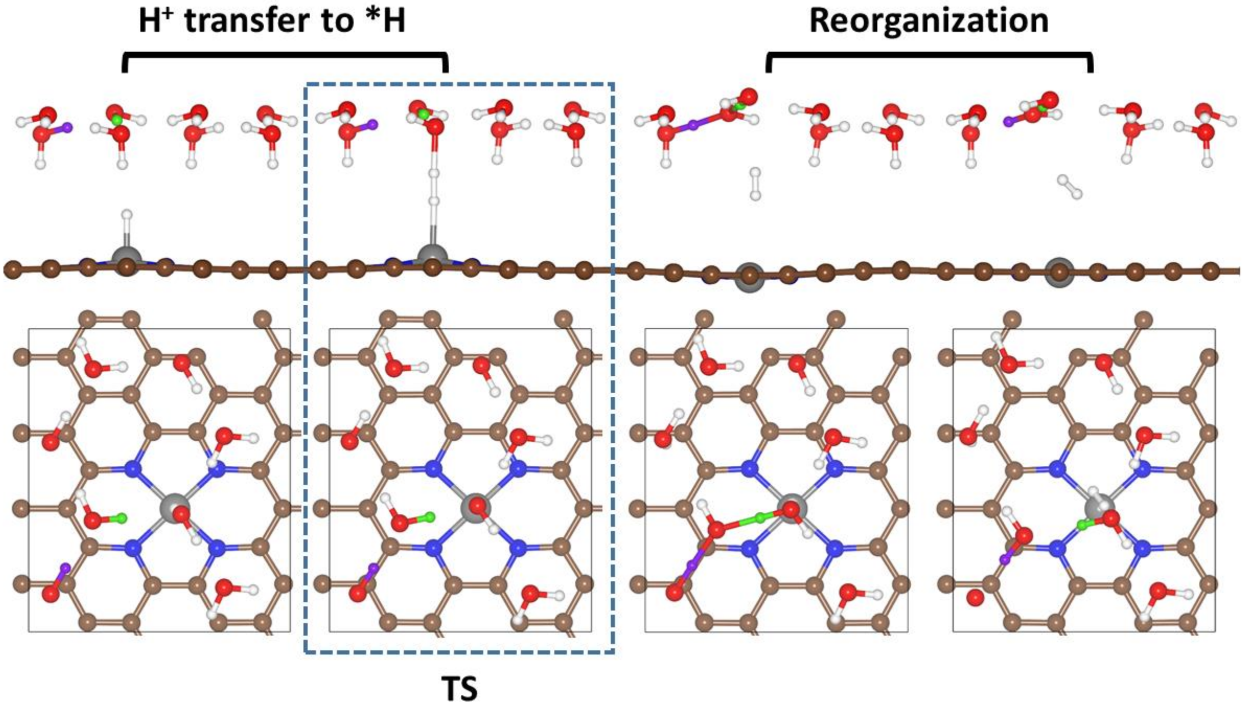

Supplementary Fig. 3 | Reaction pathway of Heyrovsky reaction under alkaline and neutral condition. Side-view and top-view are listed in the upper panel and lower panel. The transition state (TS) is highlighted with blue dashed box.

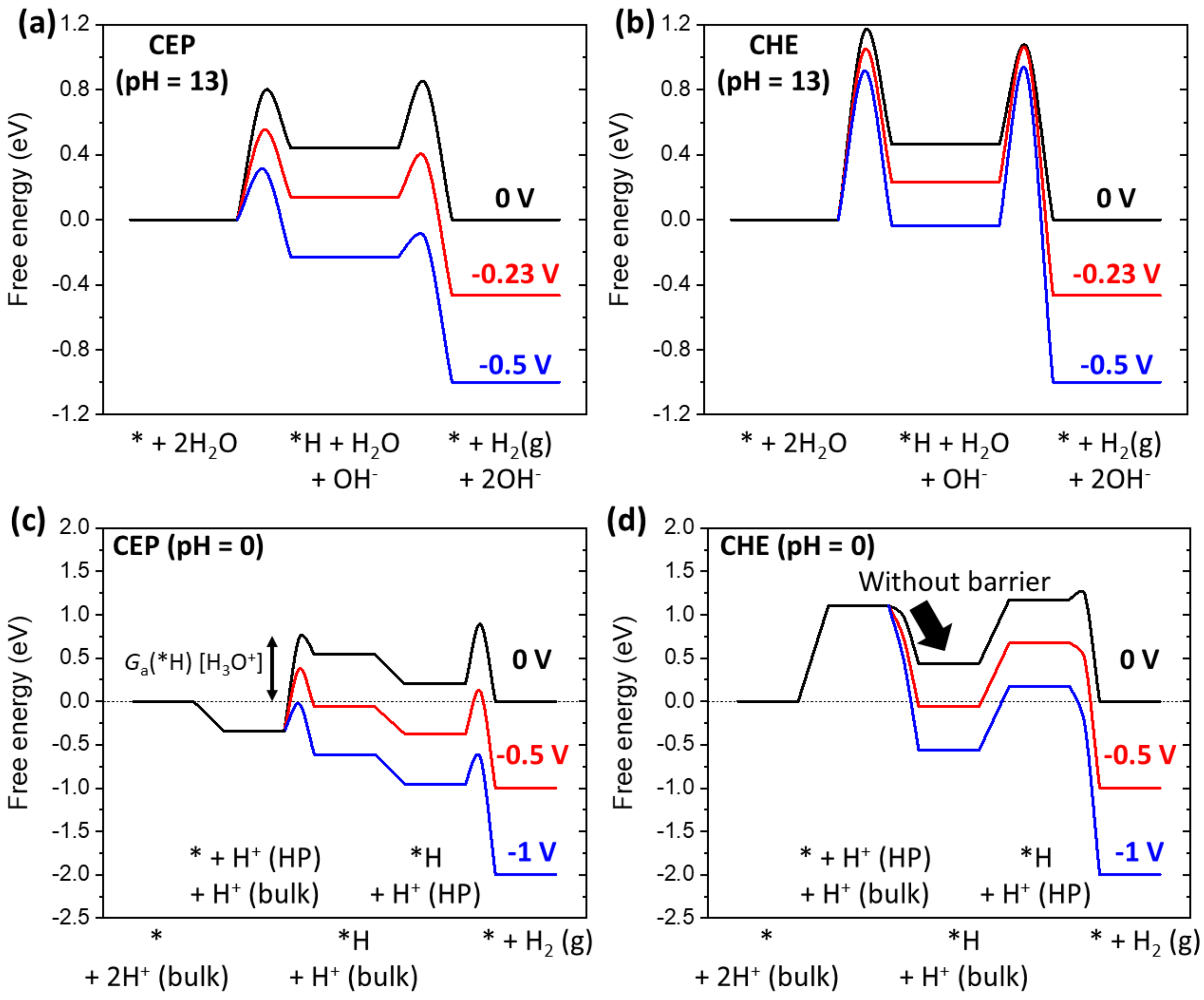

Supplementary Fig. 4 | Free energy diagram for HER on Fe@N4. Free energy diagram of HER at pH = 13 obtained by (a) the CEP model and (b) CHE model. Free energy diagram of HER at pH = 0 obtained by (c) the CEP model and (d) CHE model. All energies and U are in RHE scale at pH = 13 or pH = 0. In (d), a saddle point in the energy surface of * + H^+(HP) → *H at neutral state is not found.

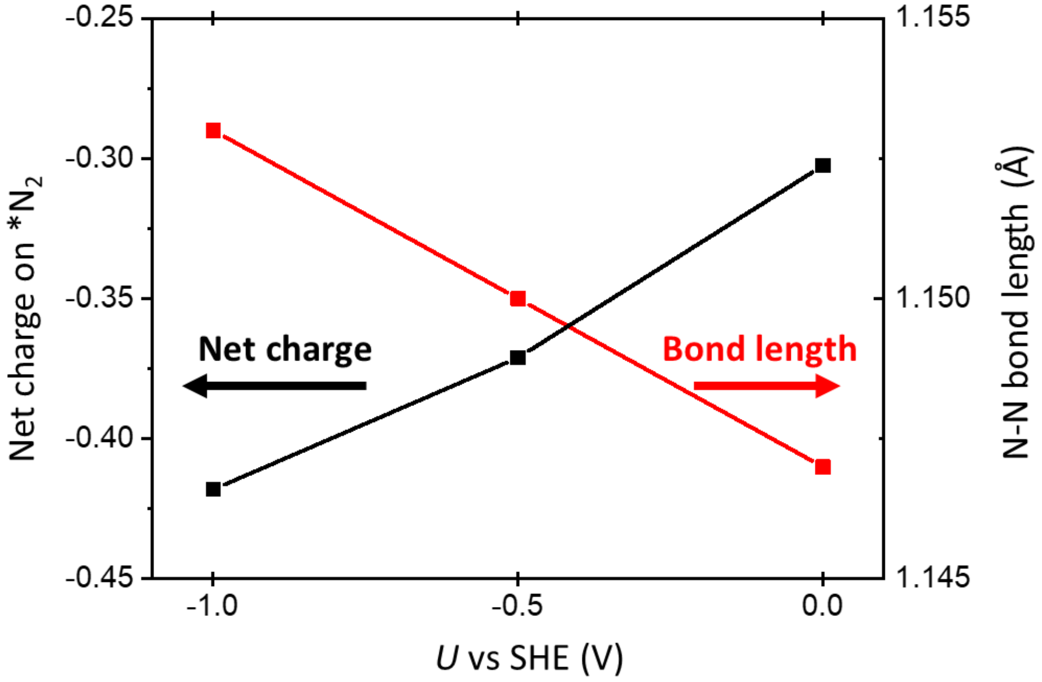

Supplementary Fig. 5 | Net charge on *N_2 and N-N bond length with *U*. The net charge in *N_2 and N-N bond length are represented by black line (left) and red line (right), respectively. The net charge is obtained by the Bader charge analysis.

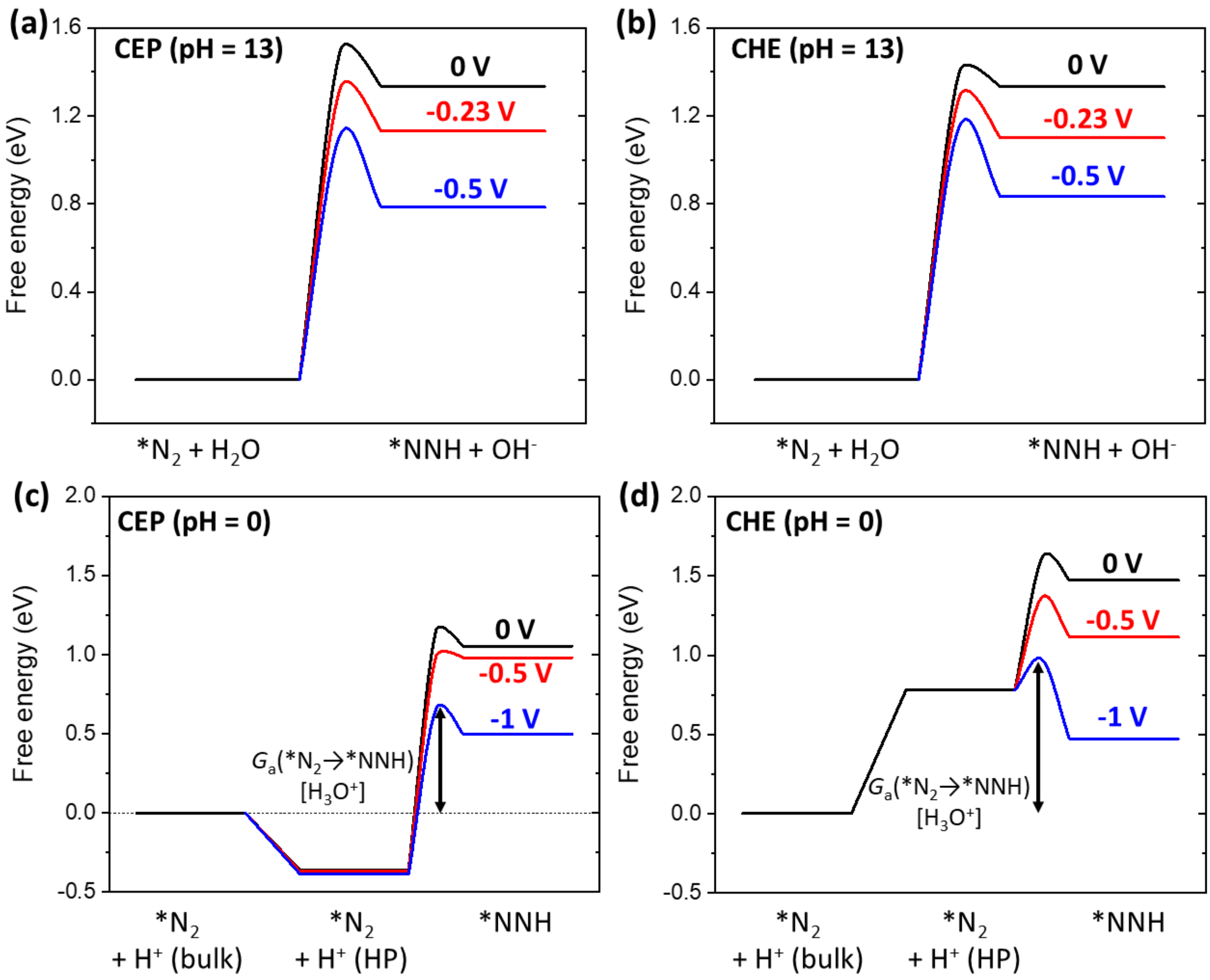

Supplementary Fig. 6 | Free energy diagram for *NNH formation. Free energy diagram for *NNH formation from *N_2 + H_2O at pH = 13 obtained by (a) the CEP model and (b) CHE model. Free energy diagram for *NNH formation from *N_2 + H^+(bulk) at pH = 0 obtained by (c) the CEP model and (d) CHE model. All energies and U are in RHE scale at pH = 13 or pH = 0.

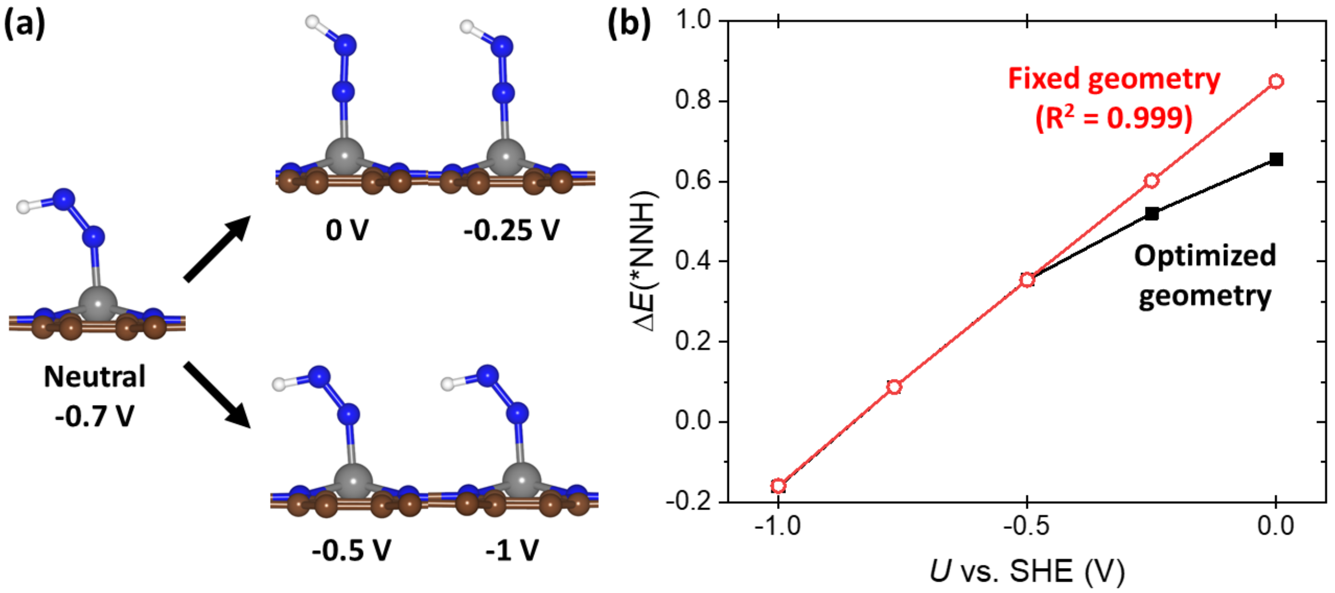

Supplementary Fig. 7 | The potential-dependent change of *NNH geometry and *NNH formation energy. (a) The optimized geometry of *NNH at neutral state (-0.7 V), 0 V, -0.25 V, -0.5 V and -1 V (vs. SHE). (b) Potential-dependent *NNH formation energy. Red line represents formation energy obtained by single point calculation using optimized structure at neutral state, while black line represents *NNH formation energy at fully optimized structure. At -0.5 V to -1 V, noticeable changes of geometry and *NNH formation energy are not observed.

Supplementary Table 3 | Data in Fig. 4a. Electrode potential and energies are in SHE scale and eV, respectively. The amount of charge transfer is in parenthesis. In this calculation, water layer is not included.

U (V)	ΔG(*H)	ΔG(*N_2)	ΔG(*$N_2 \rightarrow$*NNH)
0	0.54 (1.27)	0.40 (0.34)	1.01(0.34)
-0.25	0.23 (1.23)	0.32 (0.29)	0.96 (0.30)
-0.5	-0.06 (1.10)	0.28 (0.10)	0.83 (0.91)
-0.77	-0.33 (1.10)	0.28 (0.07)	0.56 (0.94)
-1	-0.61(1.28)	0.24 (0.28)	0.36 (0.89)

Supplementary Table 4 | Data in Fig. 4b. The amount of charge transfer in TS/FS is in parenthesis.

U (V)	ΔG_a(*H) [H_3O^+]	ΔG_a(*N_2→*NNH) [H_3O^+]	ΔG_a(*H) [H_2O]	ΔG_a(*N_2→*NNH) [H_2O]
0	0.74 (0.78 / 1.14)	1.15 (0.30[a)] / 0.31)		
-0.25	0.55 (0.72 / 1.12)	1.09 (0.32[a)] / 0.32)		
-0.5	0.37 (0.69 / 1.05)	0.98 (0.53[a)] / 0.53)		
-0.77			0.79 (0.99 / 1.19)	1.50 (0.72 / 0.96)
-1	-0.03 (0.84 / 1.22)	0.65 (0.79 / 0.90)	0.55 (1.02 / 1.35)	1.32 (0.76 / 1.12)
-1.27			0.31 (0.67 / 1.19)	1.11 (0.75 / 1.37)

[a)] Charge transfer in TS is almost same with that of FS.

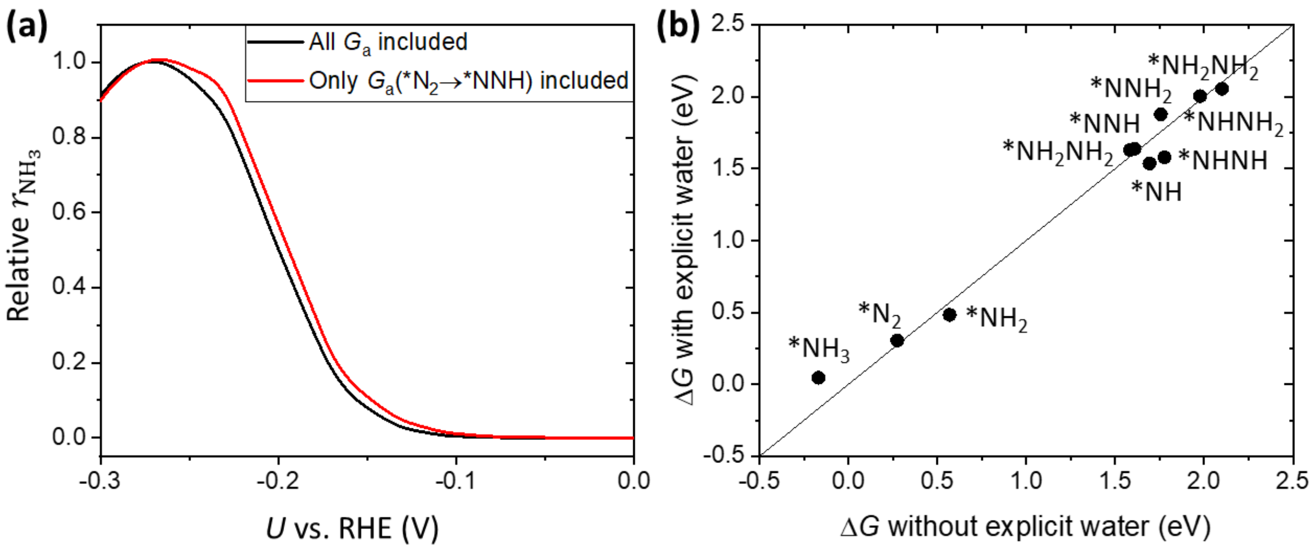

Supplementary Fig. 8 | Effect of G_a(*N_2→*NNH) and explicit water layer. (a) NH_3 yield rate obtained by MKM simulations under alkaline conditions (pH = 13). Black and red lines represent MKM result including all Ga included and that including only G_a(*N_2→*NNH). (b) Comparison of ΔG of NRR intermediates with and without explicit water layer at 0 V vs. RHE at pH = 13.

Supplementary Note 3. Potential-dependent ΔΔ*G* and ΔΔ$G_a^‡$.

The G_a(*N_2→*NNH) - G_a(*H) and Δ*G*(*N_2→*NNH) - Δ*G*(*H) increases with more negative potential (**Supplementary Fig. 9** and **Supplementary Fig. 10**). This result indicates that the difference between reaction rates of NRR (r_{NRR}) and that of HER (r_{HER}) would increase with more negative potential. The decrease of relative r_{NRR} compared to r_{HER} thus also contributes to the decrease in faradaic efficiency with more negative potential, in addition to decreasing N_2 coverage.

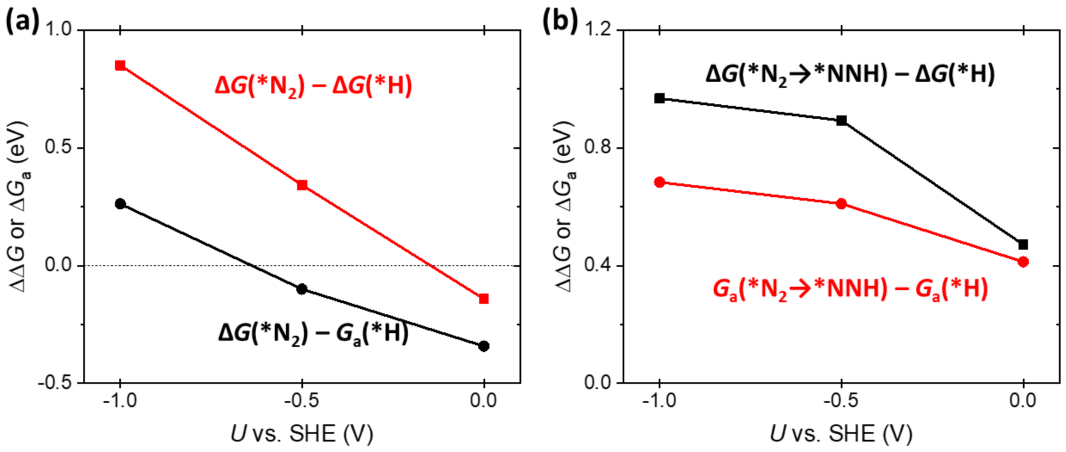

Supplementary Fig. 9 | Potential-dependent changes in ΔΔ*G* and ΔG_a under acidic condition. Changes in (a) Δ*G*(*N_2) - G_a(*H) and Δ*G*(*N_2) - Δ*G*(*H) and (b) G_a(*N_2→*NNH) - G_a(*H) and Δ*G*(*N_2→NNH) - Δ*G*(*H) by *U* under acidic condition. Solvated H_3O^+ is used as a proton donor.

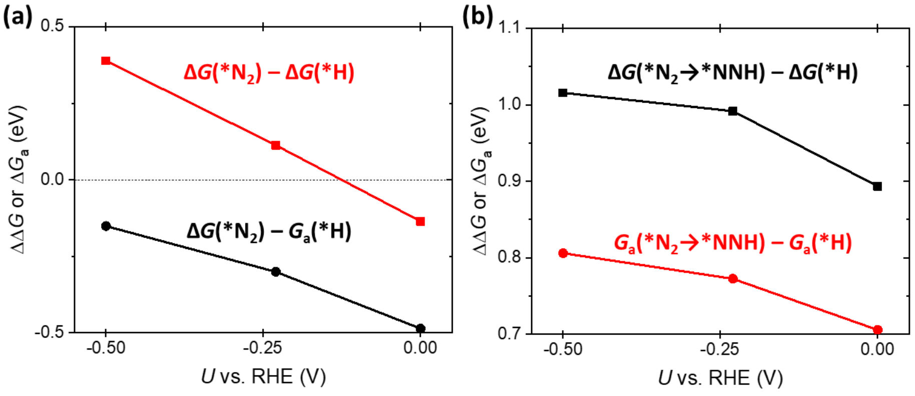

Supplementary Fig. 10 | Potential-dependent changes in ΔΔ*G* and ΔG_a under alkaline condition. Changes in (a) Δ*G*(*N_2) - G_a(*H) and Δ*G*(*N_2) - Δ*G*(*H) by *U* and (b) Changes in G_a(*N_2→*NNH) - G_a(*H) and Δ*G*(*N_2→*NNH) - Δ*G*(*H) by *U* under alkaline condition. All energies and *U* are in RHE scale at pH = 13. H_2O is used as a proton donor for obtaining activation energy.

Supplementary Note 4. Origin for deviation of the CEP model from the CHE model.

We find a deviation of the slope (Fig. 4) and reaction energy (Supplementary Fig. 11a-b) from the CEP model. Specifically, the Δ*G* for *NHNH + (H^+ + e^-) *$NHNH_2$ is 0.33 eV in the CHE model while that in the CEP model is -0.30 eV (Supplementary Fig. 11a-b). The main physical origin for such a deviation from the CHE model, we suggest, is the change in the potential of zero charge (U_{PZC}) during chemical reaction. To verify it, we compare the change in U_{PZC} after adsorption (ΔU_{PZC}) with $\Delta\Delta G_{CEP\text{-}CHE}$. Here, $\Delta\Delta G_{CEP\text{-}CHE}$ represents Δ*G* of adsorption obtained by the CEP model – Δ*G* obtained by the CHE model at 0 V (vs. SHE). The Δ*G* of each adsorbate is obtained by **(1)-(11)** in the Supplementary Note 2. We also compare ΔU_{PZC} with the deviation of slope. The deviation of the slope is obtained by slope obtained by the CEP model – slope obtained by the CHE model. We note that the slope in the CHE model is equal to the number of the transferred electrons in each Δ*G*, while the slope in the CEP model is obtained by linear regression of Δ*G* at 0 V, -0.5 V and -1 V vs. SHE (Supplementary Table 2).

A linear correlation of ΔU_{PZC} with $\Delta\Delta G_{CEP\text{-}CHE}$ as well as slope was obtained, indicating that the discrepancy originates from the change of U_{PZC} during reaction (Supplementary Fig. 12 and Supplementary Fig. 13). Now, the large discrepancy in the reaction energy of *NHNH + (H^+ + e^-) → *$NHNH_2$ can be explained. The ΔU_{PZC}(*NHNH) and ΔU_{PZC}(*$NHNH_2$) is -0.03 V and -0.26 V, respectively. Thus, the $\Delta\Delta G_{CEP\text{-}CHE}$(*NHNH) (-0.09 eV) is much closer to 0 than $\Delta\Delta G_{CEP\text{-}CHE}$(*$NHNH_2$) (-0.71 eV), leading to the large discrepancy in *NHNH → *$NHNH_2$ step between the CEP model and CHE model.

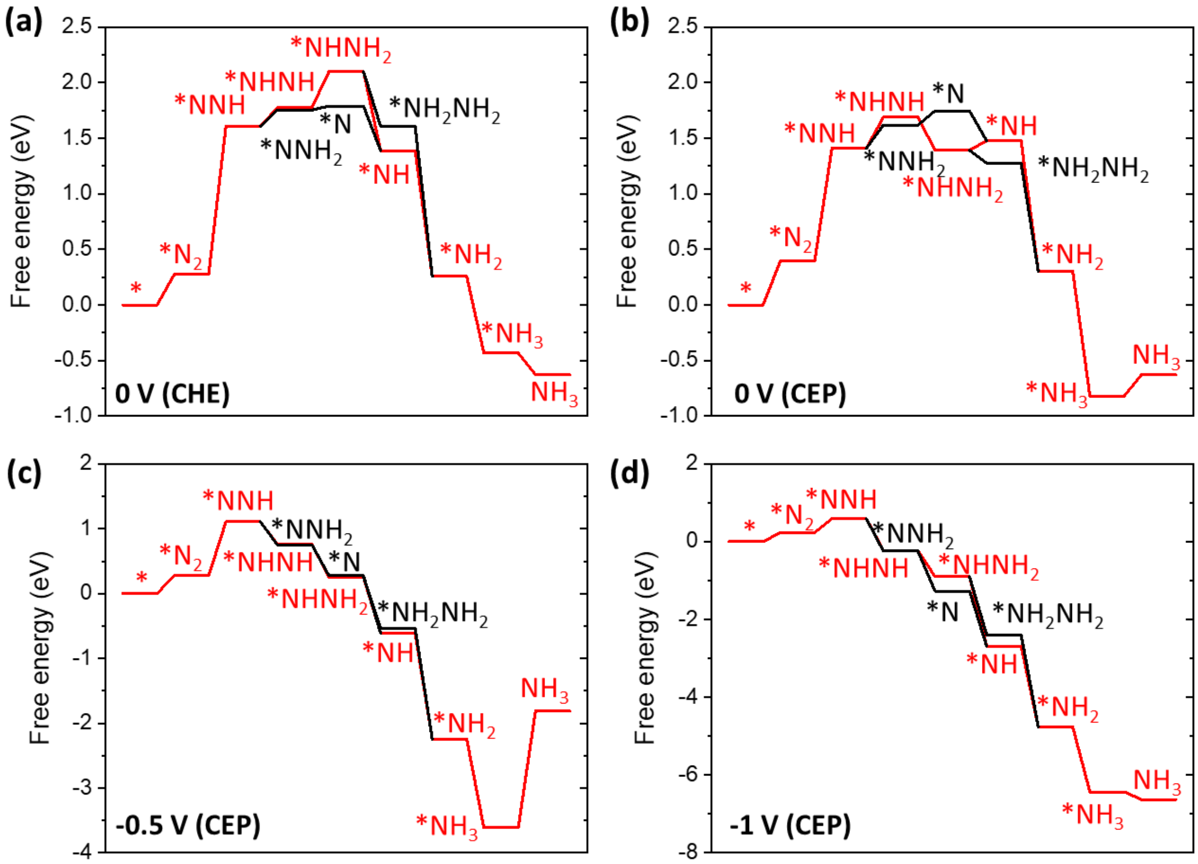

Supplementary Fig. 11 | Free energy diagram of NRR. (a) Free energy diagram of NRR on $Fe@N_4$ obtained by the CHE model at 0 V. Free energy diagram obtained by the CHE model at (b) 0 V, (c) -0.5 V and (d) -1 V (vs. SHE). The red line represents the lowest activation energy requiring reaction pathway under alkaline condition. In this calculation, the explicit water layer is not included. All energies and U are given in SHE scale at pH = 0.

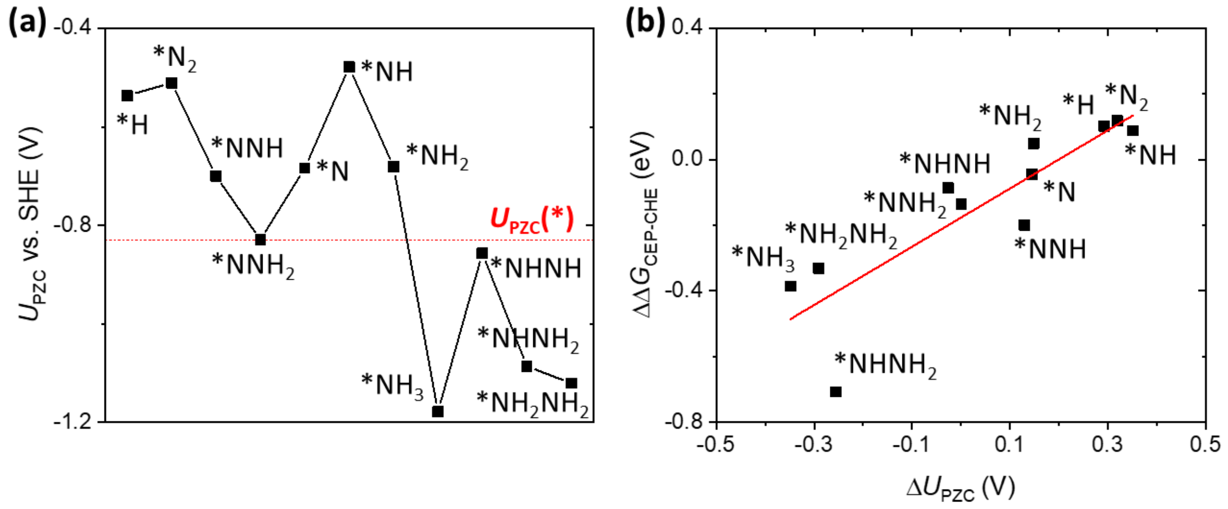

Supplementary Fig. 12 | Change of U_{PZC} by adsorption and $\Delta\Delta G_{CEP\text{-}CHE}$. (a) U_{PZC} vs. SHE (V) of each adsorbate. Horizontal red dashed line represents U_{PZC} of vacant $Fe@N_4$ site. (b) Relationship between ΔU_{PZC} and $\Delta\Delta G_{CEP\text{-}CHE}$. The $\Delta\Delta G_{CEP\text{-}CHE}$ is obtained by ΔG obtained by the CEP model - ΔG obtained by the CHE model at 0 V (vs. SHE).

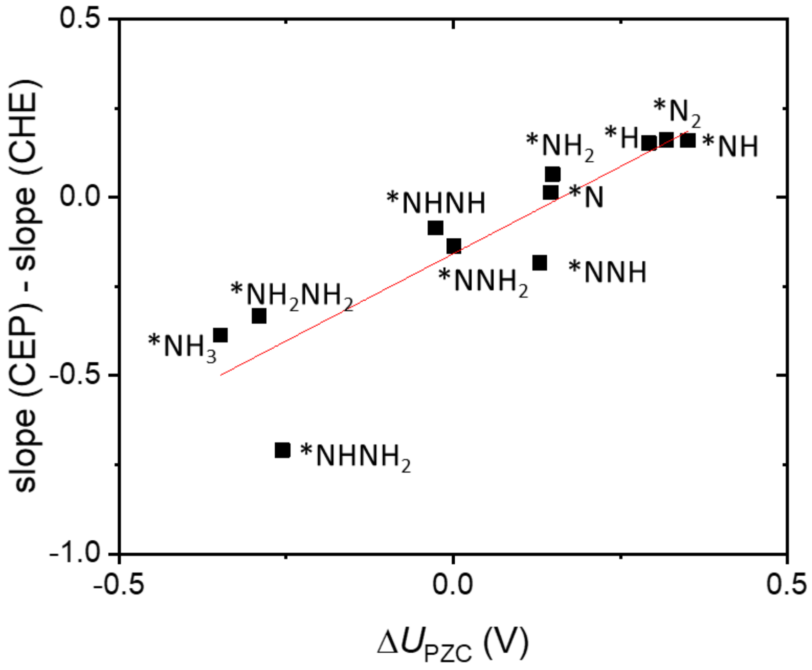

Supplementary Fig. 13 | Relation of ΔU_{PZC} with slope on Fe@N$_4$.

Supplementary Note 5. Reaction pathway for HER on Fe@N_4.

Possible HER mechanism is summarized in Supplementary Fig. 14. The Tafel reaction proceeds with two different pathway. One is the reaction of *H (H at Fe atom) with *-NH (H at adjacent N atom) and the other is the reaction of *2H (two H atoms adsorbed at Fe atom). We first find that the *H is highly more stable than *-NH by 0.92 eV at 0 V (vs. RHE at pH = 13), and hence, we exclude *-NH formation (Supplementary Fig. 15a). Next, we compared activation energy for the Heyrovsky reaction (*H + H_2O → H_2(g) + OH^-) with that of the formation of *2H (*H + H_2O → *2H + OH^-). At 0 V, the Heyrovsky reaction is kinetically preferred than *2H formation by 0.34 eV (Supplementary Fig. 15b). Thus, we conclude that the Volmer-Tafel reaction is less active than the Volmer-Heyrovsky reaction.

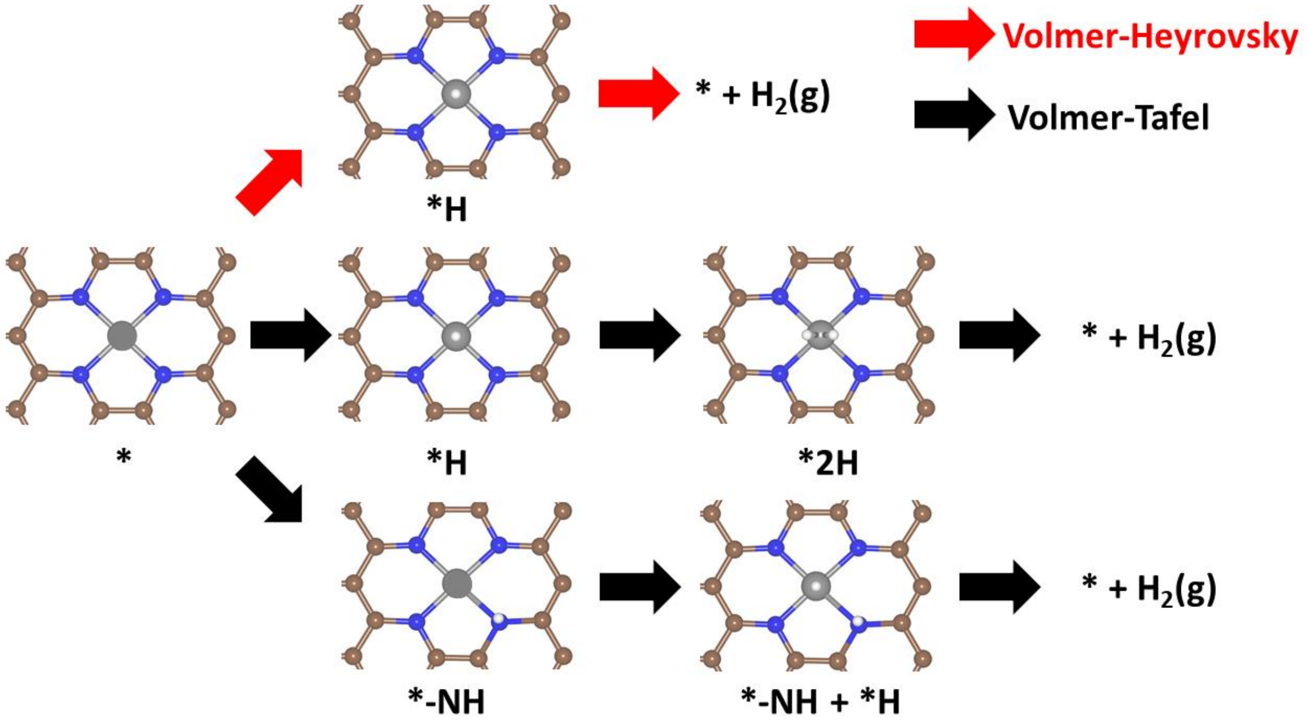

Supplementary Fig. 14 | Possible reaction pathways for HER on Fe@N_4. Grey, brown, blue and white balls represent Fe, C, N and H atom, respectively. *H and *-NH represents H adsorbed on Fe and adjacent N atom to Fe, respectively.

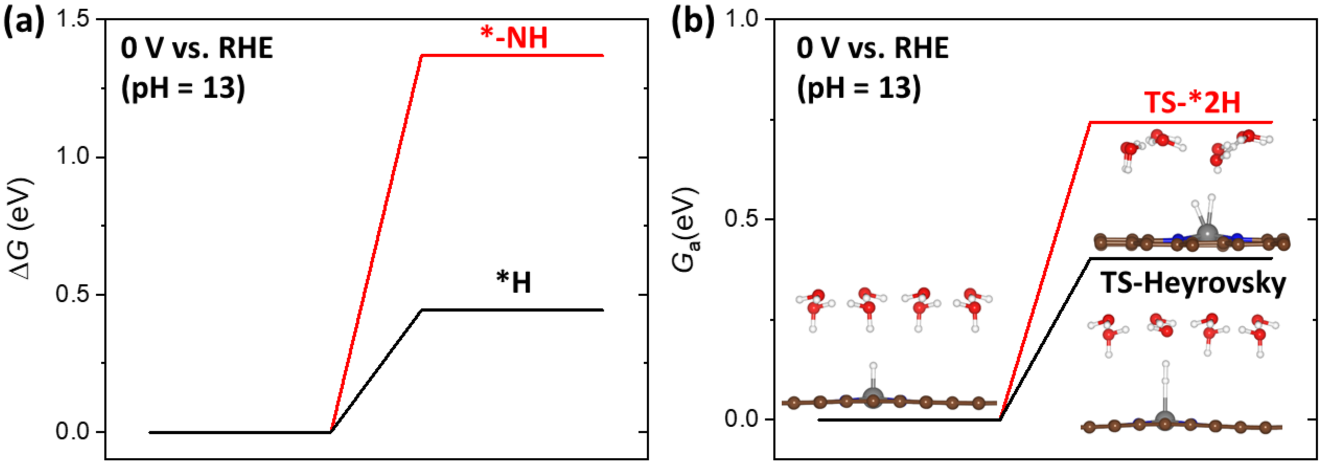

Supplementary Fig. 15 | Comparison of the Volmer-Heyrovsky pathway with the Volmer-Tafel pathway. (a) Formation free energy for *H and *-NH. (b) Activation free energy for *2H formation (*H + H_2O → *2H + OH^-) and Heyrovsky reaction (*H + H_2O → H_2(g) + OH^-). All energies are represented at 0 V vs. RHE under pH = 13. Grey, brown, blue, red and white balls represent Fe, C, N, O and H atom, respectively.

Supplementary Note 6. Microkinetic Modeling

In Wang *et al*.[12] electrochemical ammonia synthesis is performed with Fe-N-C where Fe is a single atom site. The electrochemical measurement is performed in an N_2 gas flow and water batch system, where N_2 gas is bubbled in 30 mL liquid, and the excess pressure in the gas is flown out of the system for sampling. To model this system, we set up the reactor as a mix of a continuous stirred tank reactor (CSTR) and a batch reactor. The gas is simulated in a CSTR which is in contact with the liquid in the batch reactor as shown in Supplementary Fig. 16. Both CSTR and batch control volume follows the conservation equation:

$$\rho \frac{\partial x}{\partial t} + \rho \boldsymbol{u} \cdot \nabla x = -\nabla \cdot \boldsymbol{J} \qquad \textbf{(19)}$$

Here, *x* is an extensive property such as mass of species, rho is the density, ***u*** is the velocity vector, and ***J*** is the flux vector. All constituting species in the CSTR and batch follow this equation, and simplified accordingly and solved using MatLAB ODE solver. In the synthesis, 1 mg of the catalyst is with 1.09 wt% Fe. We assumed that each Fe atom is a single atom catalyst as evidenced by high-angle annular dark-field scanning transmission electron microscopy (HAADF-STEM) image. The reaction is performed at 298 K with the N_2 gas velocity of 1 cm^3/s and 1 bar of pressure at pH = 13 as in the experiment. The volume of the gas compartment (CSTR) is 10 mL. The synthesized ammonia is set to accumulate in liquid, the chemical potential of which is calculated using the vapor pressure of ammonia and ammonia gas energetics calculated using DFT. The list of species and reactions are shown in Supplementary Table 2. We also included the water autoionization reaction with a rate constant of 2.4×10^{-2} mol m^{-3} s^{-1}. We compute the yield following the procedure described in Wang *et al*[12]. For Lü *et al.*[13], we also assume that every Fe in Fe/NC is a single atom catalytic site as HAADF-STEM image suggests. For the electrochemical measurement, 4.2 wt% Fe 1 mg catalyst is loaded to 1 cm^2 carbon paper. Here, H-type electrochemical cell is used where the configuration can be assumed the same as in Supplementary Fig. 10. The reaction is performed at 298 K and pH = 7.2, with continuously fed 1 bar N_2. We assumed that the gas and liquid control volumes are the same as it was not specified. Since the NRR measurement

under the acidic condition has not been reported, we use same reaction conditions reported by Wang *et al.*[12] for acidic conditions, but the proton donor and pH are changed into H_3O^+ and 0, respectively.

We compute the reaction constants using the transition state theory and thermodynamic equilibrium:

$$k_{\mathrm{f},i} = \frac{k_{\mathrm{b}}T}{h} \exp\left(\frac{-G_{\mathrm{a,f},i}}{k_{\mathrm{b}}T}\right)$$

$$k_{\mathrm{r},i} = \frac{k_{\mathrm{f},i}}{K} \quad \textbf{(20)}$$

$$K_i = \exp\left(\frac{-\Delta_{\mathrm{r}}G_i}{k_{\mathrm{b}}T}\right)$$

where $k_{f,i}$, and $k_{r,i}$ are the forward and reverse reaction constant of reaction i, K_i is the equilibrium constant of reaction i, k_{b} is the Boltzmann constant, T is the temperature, h is the Planck constant $G_{\mathrm{a,f},i}$ is the Gibbs free energy of activation for reaction i, and $\Delta_{\mathrm{r}}G_i$ is the Gibbs free energy of reaction for reaction i. For the reactions where we have not computed transition sate, we assumed that the $G_{\mathrm{a,f},i}$ is 0 for exothermic reactions, and $\Delta_{\mathrm{r}}G_i$ for endothermic reactions. The surface reaction rate is computed using the law of mass action:

$$r_i = k_{\mathrm{f},i} \prod\nolimits_j \alpha^{v_{i,j}} - k_{\mathrm{r},i} \prod\nolimits_j \alpha^{v_{i,j}} \quad \textbf{(21)}$$

Hereα is the activity of the species (e.g. pressure for gas, concentration for solvated species, and coverage for surface species), $v_{i,j}$ is the stoichiometric coeffcient for species j of reaction i . The time-dependent concentration is described as

$$\frac{d\theta_j}{dt} = \sum\nolimits_i v_{i,j}\, r_i \quad \textbf{(22)}$$

$$\frac{dN_j}{dt} = \sum_i v_{i,j}\, r_i \sigma$$

where the first equation describes the change in coverage and the second equation describes the change in the number of moles for gas, liquid and solvated species, and σ is the number of sites. The equation **(22)** is incorporated into equation **(19)** and solved using ODE solver.

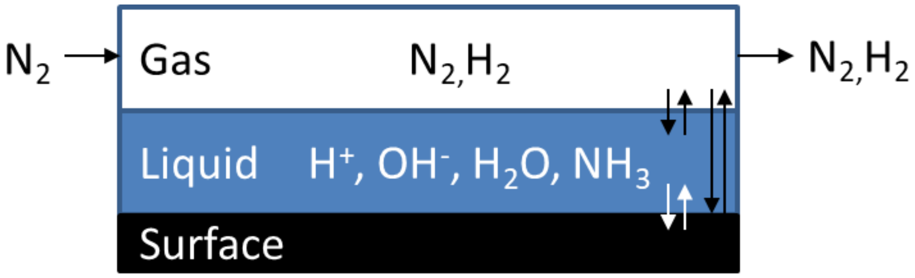

Supplementary Fig. 16 | The reactor system of the microkinetic model. Each box represents the control volume.

Supplementary Table 5 | Summary of U_{cross}, U at maximum θ_{N_2} and U at maximum NH_3 yield rate obtained on Fe@N_4 by the CEP model and MKM simulations.

pH	U_{cross} (V vs. RHE)	U at maximum θ_{N_2} (V vs. RHE)	U at maximum NH_3 yield rate (V vs. RHE)
13	-0.15	-0.175	-0.275
7.2	-0.15	-0.475	-0.575
0	-0.15	-0.15	-0.20

Supplementary Note 7. Microkinetic Modeling using the CHE model.

The reactor system and elementary reactions are same with those of MKM using the CEP model (Supplementary Note 6). For estimating the activation energy in the CHE model, the activation energy obtained at the neutral state (net charge = 0) is assumed to be a activation energy at 0 V (vs. SHE) at standard state. We corrected -0.702 eV for the solvated OH^- same in the CEP model (Supplementary Note 2).

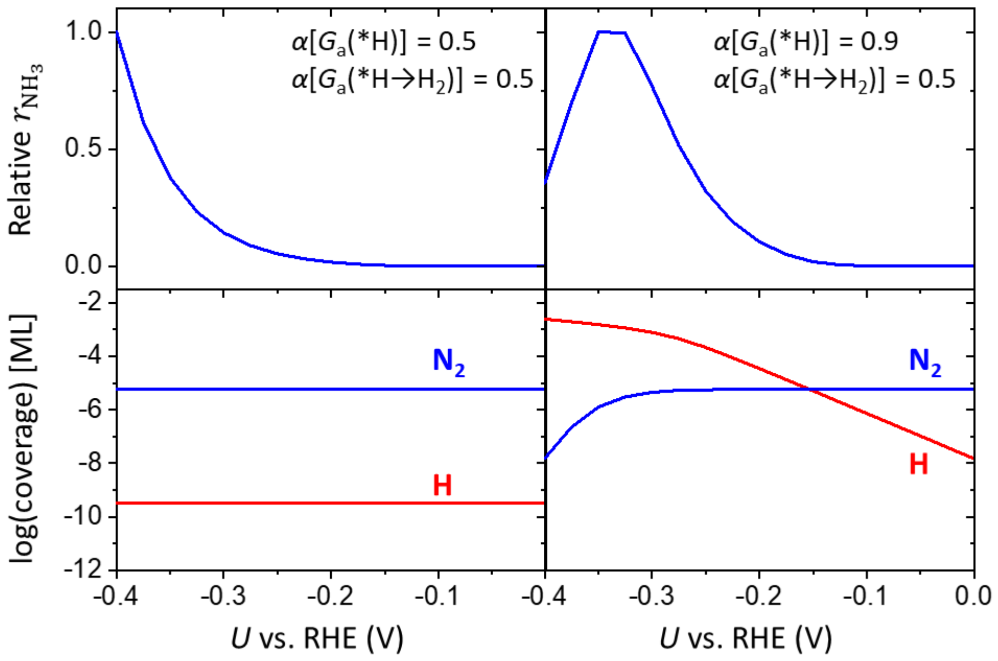

Supplementary Fig. 17 | The change in θ_{N_2}, θ_H and r_{NH_3} by U obtained by the microkinetic modeling (MKM) using the CHE model with different charge transfer in TS of *H formation. The relative r_{NH_3} and coverage are shown in upper and lower panels, respectively. Electrode potential (U) is in RHE scale at pH = 13.

Supplementary Note 8. Calculation of $\Delta\overline{N_e}$ and slope

The ΔG and ΔN_e at U = 0, -0.5, -1 V (vs. SHE) are used to obtain the calculated slope and $\Delta\overline{N_e}$, respectively. Here, we use the number of electrons added (or extracted) to the slab model to tune the potential (or workfunction) of the slab surface as N_e. The charge neutrality is kept by ionic countercharges via the linearized Poisson-Boltzmann equation.

We found that the ΔN_e does not change by explicit water significantly (< 0.1 e^-) (Supplementary Table 3-4). Hence, the explicit water layer is not included in calculating the slope and $\Delta\overline{N_e}$ on Fe(110), Ru(0001), Rh(111), Fe@N_3, Ru@NC_2, Ru@N_3 and Ag@N_4. The $\Delta\overline{N_e}$ and slope are obtained by using ΔG and ΔN_e at 0 V, -0.5 V, -1 V (vs. SHE).

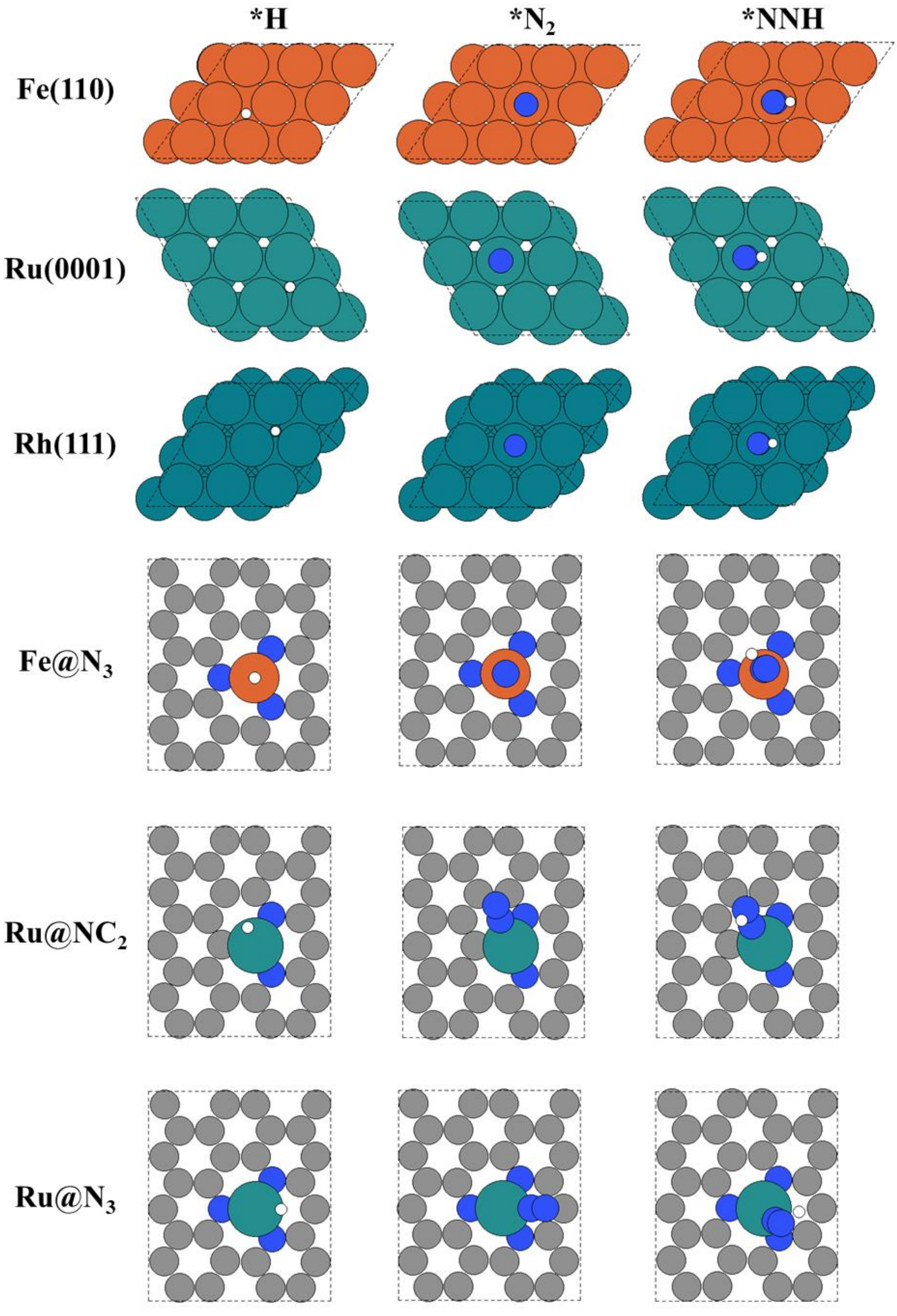

Supplementary Fig. 18 | The top-view of the optimized structures for *H, *N$_2$ and *NNH on Fe(110), Ru(0001), Rh(111), Fe@N$_3$, Ru@NC$_2$, and Ru@N$_3$. On the metal surfaces, top site adsorption is considered for *N$_2$ and *NNH, while the hollow-site is considered for *H.

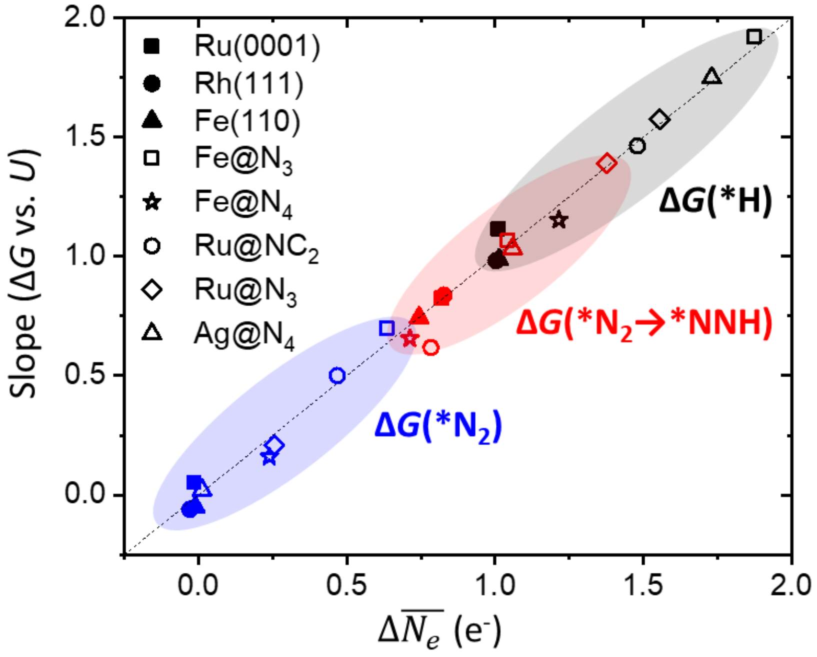

Supplementary Fig. 19 | Linear correlation between the slope (the rate of change in Δ*G* with potential) and $\Delta\overline{N_e}$ for Δ*G*(*H), Δ*G*(*N_2) and Δ*G*(*N_2→*NNH). Black, blue and red colors represent Δ*G*(*H), Δ*G*(*N_2) and Δ*G*(*N_2→*NNH), respectively. The diagonal dashed line indicates the location at slope (Δ*G* vs. *U*) = $\Delta\overline{N_e}$.

Supplementary Table 6 | The Δ*N*e and slope for Δ*G*(*H), Δ*G*(*N_2) and Δ*G*(*N_2→*NNH). The electrode potential is in V vs. SHE.

Reactions	*U* (V)	Fe(110)	Ru(0001)	Rh(111)	Fe@N_3	Fe@N_4	Ru@NC_2	Ru@N_3	Ag@N_4
* + H^+ → *H	0	1.03	1.04	1.04	1.89	1.27	1.50	1.46	1.72
	-0.5	1.01	1.00	1.01	1.88	1.10	1.47	1.63	1.68
	-1	0.99	0.97	0.99	1.85	1.28	1.46	1.58	1.79
	slope	1.12	0.98	0.99	1.92	1.15	1.46	1.57	1.75
* + N_2 → *N_2	0	0.02	-0.05	0.00	0.31	0.34	0.47	0.32	0.02
	-0.5	-0.03	-0.03	-0.02	0.79	0.10	0.58	0.22	0.04
	-1	-0.03	0.00	-0.01	0.81	0.28	0.35	0.22	-0.02
	slope	0.05	-0.06	-0.05	0.70	0.16	0.50	0.21	0.02
*N_2 + H^+ → *NNH	0	0.78	0.80	0.70	1.14	0.34	0.83	1.00	0.96
	-0.5	0.82	0.83	0.74	0.98	0.91	0.39	1.57	0.97
	-1	0.85	0.85	0.79	1.00	0.89	1.13	1.56	1.25
	slope	0.82	0.84	0.74	1.07	0.66	0.62	1.39	1.03

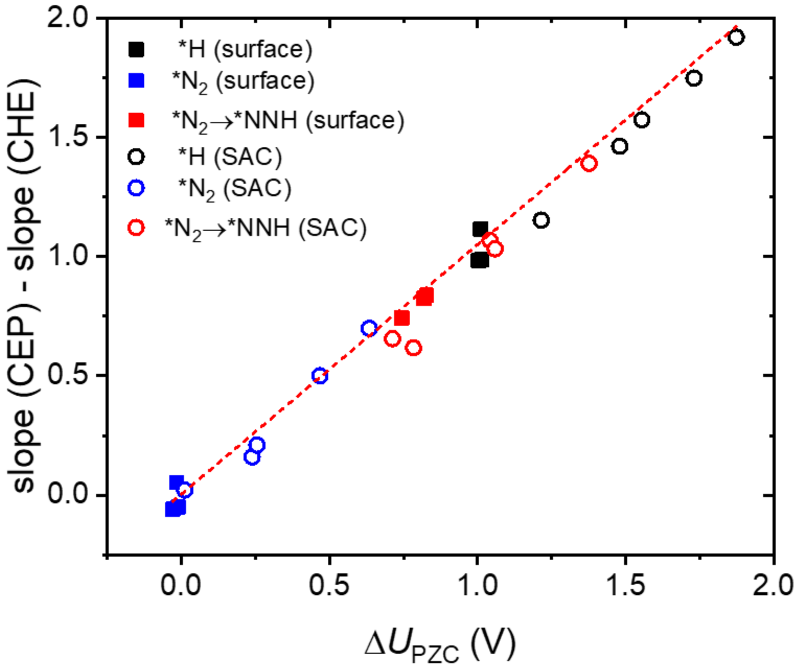

Supplementary Fig. 20 | Linear correlation between the slope and ΔU_{PZC}. Black, blue and red colors represent Δ*G*(*H), Δ*G*(*N_2) and Δ*G*(*N_2→*NNH), respectively.

Supplementary References

1. Liu, H.-M. *et al.* Surfactant-free atomically ultrathin rhodium nanosheet nanoassemblies for efficient nitrogen electroreduction. *J. Mater. Chem. A* **6**, 3211-3217 (2018).
2. Yang, D., Chen, T. & Wang, Z. Electrochemical reduction of aqueous nitrogen (N_2) at a low overpotential on (110)-oriented Mo nanofilm. *J. Mater. Chem. A* **5**, 18967-18971 (2017).
3. Hu, L. *et al.* Ambient electrochemical ammonia synthesis with high selectivity on Fe/Fe oxide catalyst. *ACS Catal.* **8**, 9312-9319 (2018).
4. Wang, D. *et al.* Energy-Efficient Nitrogen Reduction to Ammonia at Low Overpotential in Aqueous Electrolyte under Ambient Conditions. *ChemSusChem* **11**, 3416-3422 (2018).
5. Bao, D. *et al.* Electrochemical reduction of N_2 under ambient conditions for artificial N_2 fixation and renewable energy storage using N_2/NH_3 cycle. *Adv. Mater.* **29**, 1604799 (2017).
6. Tao, H. *et al.* Nitrogen fixation by Ru single-atom electrocatalytic reduction. *Chem* **5**, 204-214 (2019).
7. Zhang, X., Kong, R.-M., Du, H., Xia, L. & Qu, F. Highly efficient electrochemical ammonia synthesis via nitrogen reduction reactions on a VN nanowire array under ambient conditions. *Chem. Commun.* **54**, 5323-5325 (2018).
8. Yang, X. *et al.* Mechanistic insights into electrochemical nitrogen reduction reaction on vanadium nitride nanoparticles. *J. Am. Chem. Soc.* **140**, 13387-13391 (2018).
9. Ren, X. *et al.* Electrochemical N_2 fixation to NH_3 under ambient conditions: Mo_2N nanorod as a highly efficient and selective catalyst. *Chem. Commun.* **54**, 8474-8477 (2018).
10. Xu, X. *et al.* 1 T-phase molybdenum sulfide nanodots enable efficient electrocatalytic nitrogen fixation under ambient conditions. *Appl. Catal. B*, 118984 (2020).
11. Wang, Y. *et al.* Rational design of Fe–N/C hybrid for enhanced nitrogen reduction electrocatalysis under ambient conditions in aqueous solution. *ACS Catal.* **9**, 336-344 (2018).
12. Wang, M. *et al.* Over 56.55% Faradaic efficiency of ambient ammonia synthesis enabled by positively shifting the reaction potential. *Nat. Commun.* **10**, 1-8 (2019).
13. Lü, F. *et al.* Nitrogen-coordinated single Fe sites for efficient electrocatalytic N_2 fixation in neutral media. *Nano Energy* **61**, 420-427 (2019).
14. Chen, Y. *et al.* Highly Productive Electrosynthesis of Ammonia by Admolecule-Targeting Single Ag Sites. *ACS Nano* **14**, 6938-6946 (2020).
15. wang, h. *et al.* Bionic Design of Mo (IV) Doped FeS2 Catalyst for Electroreduction of Dinitrogen to Ammonia. *ACS Catal.* **10**, 4914−4921 (2020).
16. Han, Z. *et al.* Activated TiO_2 with tuned vacancy for efficient electrochemical nitrogen reduction. *Appl. Catal. B* **257**, 117896 (2019).
17. Mukherjee, S. *et al.* Metal-organic framework-derived nitrogen-doped highly disordered carbon for electrochemical ammonia synthesis using N_2 and H_2O in alkaline electrolytes. *Nano Energy* **48**, 217-226 (2018).
18. Yu, X. *et al.* Boron-doped graphene for electrocatalytic N_2 reduction. *Joule* **2**, 1610-1622 (2018).
19. Zhang, M. *et al.* Reduced graphene oxides with engineered defects enable efficient electrochemical reduction of dinitrogen to ammonia in wide pH range. *Nano Energy* **68**, 104323 (2020).
20. Lindgren, P., Kastlunger, G. & Peterson, A. A. A Challenge to the G～ 0 Interpretation of Hydrogen Evolution. *ACS Catal.* **10**, 121-128 (2019).
21. Hörmann, N. G., Andreussi, O. & Marzari, N. Grand canonical simulations of electrochemical interfaces in implicit solvation models. *J. Chem. Phys.* **150**, 041730 (2019).
22. Nørskov, J. K. *et al.* Origin of the overpotential for oxygen reduction at a fuel-cell cathode. *J. Phys. Chem. B* **108**, 17886-17892 (2004).
23. Trasatti, S. The absolute electrode potential: an explanatory note (Recommendations 1986). *Pure and Applied Chemistry* **58**, 955-966 (1986).

24. Jinnouchi, R. & Anderson, A. B. Aqueous and surface redox potentials from self-consistently determined Gibbs energies. *J. Phys. Chem. C* **112**, 8747-8750 (2008).
25. Lamoureux, P. S., Singh, A. R. & Chan, K. pH effects on hydrogen evolution and oxidation over Pt (111): Insights from first-principles. *ACS Catal.* **9**, 6194-6201 (2019).
26. Garcia-Ratés, M. & López, N. Multigrid-based methodology for implicit solvation models in periodic DFT. *J. Chem. Theory Comput.* **12**, 1331-1341 (2016).
27. Liu, L., Liu, Y. & Liu, C. Enhancing the understanding of hydrogen evolution and oxidation reactions on Pt (111) through ab initio simulation of electrode/electrolyte kinetics. *J. Am. Chem. Soc.* **142**, 4985-4989 (2020).
28. Gauthier, J. A., Chen, L. D., Bajdich, M. & Chan, K. Implications of the fractional charge of hydroxide at the electrochemical interface. *Phys. Chem. Chem. Phys.* **22**, 6964-6969 (2020).
29. Perman, E. P. CXV.—Vapour pressure of aqueous ammonia solution. Part II. *Journal of the Chemical Society, Transactions* **83**, 1168-1184 (1903).
